# Self-Esteem, Socioeconomic Status and Social Participation of Persons with Disabilities Living in Areas Affected by Armed Conflict in Colombia

**DOI:** 10.3390/ijerph18084328

**Published:** 2021-04-19

**Authors:** Takuya Yamada, Kayako Sakisaka, Lady Nayibe Bermúdez Rodríguez, Kazue Yamaoka

**Affiliations:** 1 School of Public Health, Teikyo University Graduate, Tokyo 173-8605, Japan; kazue@med.teikyo-u.ac.jp; 2 Tsudoinooka Home-Visit Nursing Care Station, Eishinkai Medical Corporation, Shizuoka 420-0066, Japan; 3 Grupo de Enfoques Diferenciales, Unidad para la Atención y Reparación Integral a las Víctimas, Bogotá 111071, Colombia; lady.bermudez@unidadvictimas.gov.co

**Keywords:** armed conflict, persons with disabilities, social participation, self-esteem, Colombia

## Abstract

This cross-sectional study explored the association between self-esteem and social participation of persons with disabilities living in two municipalities affected by armed conflict in Colombia. We studied the socioeconomic status, communication level, social participation, and self-esteem of the participants. The Rosenberg Self-Esteem Scale (RSES) was used to evaluate the level of self-esteem. We performed bivariate analysis and multiple regression analysis to identify the determinants of higher self-esteem in the target populations. In total, there were 579 participants in the study. The mean RSES score was 28.8 (SD = 4.5). Self-esteem was associated with monthly household income (β = 0.45, *p* = 0.028), education level (β = 0.65, *p* = 0.048), current job (β = 1.00, *p* = 0.017), type of disability (β = −1.17, *p* = 0.002), frequency of communication with neighbors or friends (β = 0.53, *p* = 0.013), and participation in community organization activities (β = 0.89, *p* = 0.019). Frequent communication with their own community, higher levels of school education, and having a job were determinants of higher self-esteem in persons with disabilities. We suggest the importance of an active inclusive reconstruction program to support persons with disabilities in local municipalities affected by armed conflict in developing countries.

## 1. Introduction

Since World War II, the world has faced many types of armed conflict, colonial wars, wars for national liberation, cold war-related struggles, religious conflicts, political conflicts, and more [1]. Between 1946 and 2019, 334 episodes of armed conflict broke out in the world [2]. As a result of these numerous conflicts, the number of people living in areas affected by armed conflict has increased to about 6% of the world’s population [3]. Armed conflicts leave human life in chaos and lead to public health problems [4]. Charlson et al. [5] estimated that 22.1% of the population affected by armed conflict have mental disorders. Persons with disabilities are especially greatly affected by armed conflict and are vulnerable to humanitarian crises [6]. The World Health Organization estimates that more than 1 billion people in the world, almost 15%, live with disabilities [7], and of these, 160 million are attributable to armed conflict [8]. The impact of armed conflict on persons with disabilities is enormous.

The Republic of Colombia (Colombia) is known as one of the countries affected by armed conflict, which has continued over five decades between the government armed forces, anti-government guerrillas, and paramilitaries [9]. The internal conflicts in Colombia have caused extensive damage. According to the national data, the total number of armed conflicts was reported to be more than 10 million, and the total number of victims of armed conflict was more than eight million [10]. The Colombian government has engaged in efforts to terminate conflicts with anti-government guerrillas for many years, and has conducted post-conflict programs to reconstruct the areas affected by armed conflict, and to provide relief to victims.

The government has estimated that approximately 6.4% of the total population in Colombia are persons with disabilities [11]. Furthermore, 4.4% of victims of armed conflict are left as victims with disabilities [11]. However, the actual situation of persons with disabilities living in rural areas who have been affected by armed conflict has not received much research attention. Inaccessible infrastructure, lack of information and communication, stigma from the community, and complex service systems has impeded relief programs from reaching them [7]. Therefore, persons with disabilities owing to armed conflict tend to be isolated in rural areas and left behind by relief programs. As such, it is unknown whether their self-esteem is affected by their unfortunate circumstances.

Rosenberg defined that self-esteem is a positive or negative attitude an individual has toward themselves [12]. A previous study has suggested that self-esteem in persons with physical disabilities might be lower than that of peers without disabilities [13]. Self-esteem is associated with life satisfaction [14,15,16,17], quality of life [18], psychological health [15,19,20,21], interpersonal and social relationships [13,22], and employment [1,13,16,20]. In persons with disabilities, self-esteem might be mediated and moderated by stigma perception and social relationships [22]. Moreover, self-esteem influences the resilience to adversity of persons with spina bifida [19]. The predictors of self-esteem are reported to be socioeconomic status [17,23,24] and support from family members or others [13,25]. Among vulnerable people, such as refugee women and persons with disabilities, social and family treatment [13,25] might be a predictor of level of self-esteem. These results were based on data from individuals without disabilities or persons with disabilities living in a city area or having access to a support center. Socioeconomic status, participation, and self-esteem of persons with disabilities in local municipalities affected by armed conflict have not been investigated. Accordingly, it is important to clarify their self-esteem and associated factors for improving their self-esteem to develop an inclusive relief program at post-conflict areas.

The purposes of this study were as follows: (1) describe the socioeconomic status, social participation, and self-esteem of persons with disabilities affected by armed conflict in rural Colombia and (2) identify the determinants of higher self-esteem.

## 2. Materials and Methods

### 2.1. Study Site and Target Population

**Figure 1:**
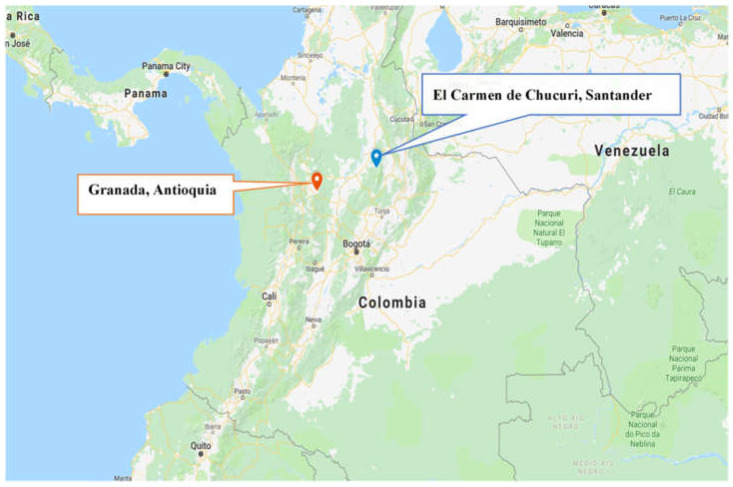
Location of study sites (Google Maps).

Colombian Government Unit for comprehensive attention and reparation to victims of internal conflict and Japan International Cooperation Agency (JICA) implemented the Project for Social Inclusion of Conflict Victims with Disabilities (hereinafter referred to as “the Project”) in two local municipalities; Granada, Antioquia and El Carmen de Chucurí, Santander were the first pilot sites [26]. The Project was implemented from March 2015 to March 2020. The location of the study sites was approximately 210 km to Granada, Antioquia, and 240 km to El Carmen de Chucurí, Santander from the city of Bogota, the capital of Colombia (Figure 1). The main purpose of the Project was the implementation of social inclusion strategies for persons with disabilities living in rural areas affected by conflict. Because the situation of persons with disabilities in rural areas affected by armed conflict was not understood, the first activity of the Project was to conduct a survey to collect baseline data. Based on the survey data, the Project designed activities such as raising awareness about disabilities and increasing accessibility.

The baseline survey included information on the current life conditions, socioeconomic status, activities of daily living, and level of participation in society for victims and non-victims with disabilities. The survey was conducted from September to November 2015.

**Figure 2:**
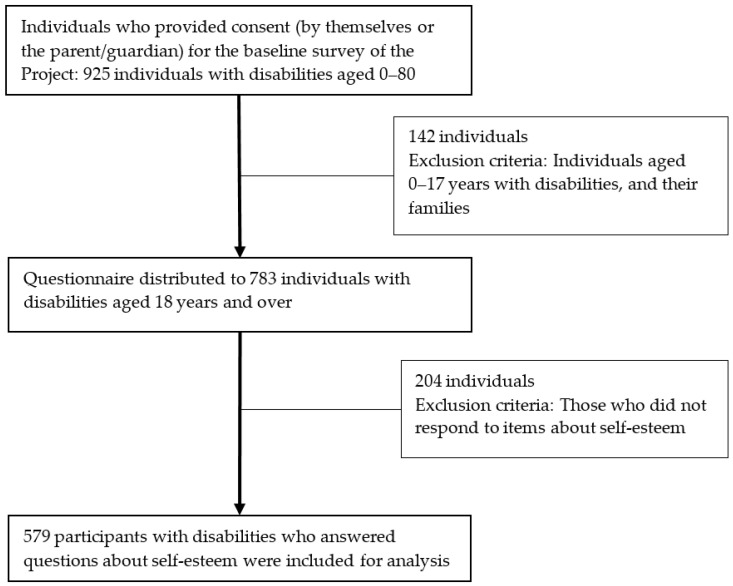
Flow of the study.

The participants in the Project’s baseline survey were all persons with disabilities aged 0–80 years residing in the two Project sites. They were chosen from the list of persons with disabilities that the local governments had and from the information of the people in the local district. Staff from the Project and local government contacted participants by mobile phone to schedule a date for administering the questionnaire in a face-to-face interview. A total of 925 persons with disabilities (Granada: 510; El Carmen de Chucuri: 415) participated in the survey after obtaining informed consent. We excluded 142 persons with disabilities aged 0–17 years from the analyses because a family member participated in the interview on behalf of the child. Consequently, the responses to the items would not be those of the child. In addition, we excluded 204 participants aged 18 and older who did not respond to items about self-esteem. The final sample consisted of 579 persons with disabilities aged 18–80 years (Figure 2).

### 2.2. Study Design

This was a cross-sectional study using a structured questionnaire with face-to-face interviews conducted by trained local interviewers. The project implemented data collection in two local municipalities affected by armed conflict.

### 2.3. Measurements

To measure the level of self-esteem, we used the Rosenberg Self-Esteem Scale (RSES). The RSES was developed by Rosenberg [12] to assess a person’s evaluation of a positive or negative attitude toward the self. The RSES has ten items that are rated on a 4-point Likert scale (1 = strongly disagree, 2 = disagree, 3 = agree, 4 = strongly agree). The total score ranged from 10 to 40 points; higher scores indicated better self-esteem. The RSES has been translated into various languages. The project adopted the Spanish version of the RSES to fit Colombia’s rural situation (Cronbach α = 0.73).

Regarding socioeconomic status, we selected 10 items from the responses to the structured questionnaire: age, sex, monthly household income, education level, reading ability (Yes/No), writing ability (Yes/No), residential area (Urban/Rural), current work (Yes/No), type of disability, and victim of armed conflict (Yes/No). Type of disability was self-reported because there was no documentation or certification of a medical diagnosis indicating type of disability. Participants chose from six options on the questionnaire: physical disability, hearing disability, visual disability, intellectual disability, psychosocial disability, and do not know. If they chose multiple options, they were considered as multiple disabilities. There were five options for monthly household income: none, low, lower middle, upper middle, and high. High income was more than the minimum wage in 2015 in Colombia [700,001 COP (about 226 US dollars)] [27,28]. Education level was divided into three levels: none or pre-school level, basic primary, and secondary school or above.

To analyze social participation, we chose four items from the responses to the structured questionnaire: frequency of communication with family members, frequency of communication with neighbors or friends, going out from home (Yes/No), and level of participation in community organization activities (Yes/No). Frequency of communication with family members and with neighbors or friends had three answers: every day, some days a week, occasionally or not communicate (Table S1 provides the socioeconomic status and social participation items with their response options).

### 2.4. Statistical Analysis

First, we calculated descriptive statistics for all the variables of interest in the study. Descriptive statistics included means and standard deviations for continuous variables, and frequencies and percentages for categorical variables. Second, means and standard deviations of the RSES scores were calculated for each independent variable category. Age was divided into three categories: under 40 years, 40–60 years, and over 60 years. To examine the raw associations of socioeconomic status and other possible factors with self-esteem, we conducted a univariate regression analysis. Then, we performed multiple regression analysis including all socioeconomic and social participation variables to identify determinants of the higher RSES scores using the stepwise variable selection method (inclusion criteria ≤ 0.20 and exclusion criteria > 0.20). Statistical significance was tested with a two-tailed test of significance at *p* < 0.05. SAS Version 9.4 (SAS Institute, Cary, NC, USA) was used for all statistical analyses.

### 2.5. Ethical Considerations

This study analyzed the data from the reports published by the Project for Social Inclusion of Conflict Victims with Disabilities, Colombia [29]. We adopted secondary data from the above study, and all personal information was previously converted to a blinded number. The authors did not access any personal information except for questionnaire answers from each respondent; therefore, an additional ethical clearance procedure was not applied to this study.

## 3. Results

### 3.1. Sociodemographic Characteristics and Social Participation of Participants

Table 1 summarizes the sociodemographic characteristics and social participation of the 579 participants. A total of 252 participants lived in El Carmen de Chucuri, and 327 participants lived in Granada. Of these, 78.9% were victims of armed conflict, 44.9% were female, and 13.6% had not participated in basic primary education. The mean age was 51.9 years. The types of disabilities included physical (42.8%), multiple (18.0%), visual (11.9%), psychosocial (10.5%), intellectual (6.4%), and hearing (3.1%). Some participants (7.3%) responded with “do not know” for type of disability. Occasional or no communication with neighbors or friends was reported by 52.7%, and 38.9% participated in community organization activities.

### 3.2. Associations between Socioeconomic, Social Participation, and the RSES Score

Table 2 shows the mean score of RSES by socioeconomic status and social participation, and the results of the univariate regression analysis. The mean RSES score was 28.8 (SD *=* 4.5) for all participants. The variables related to monthly income (*p* = 0.002), education level (*p* < 0.001), including reading ability (*p* = 0.008) and writing ability (*p* = 0.002), living in urban areas (*p* = 0.011), and social participation, such as having a job (*p* = 0.009), frequent communication with neighbors or friends (*p* < 0.001), frequently going outside (*p* < 0.001), and participation in community organization activities in the last six months (*p* = 0.002) were significantly associated with the RSES scores. This revealed that persons with disabilities with a higher education level and frequency of communication with neighbors or friends were more likely to have higher self-esteem.

The results of the stepwise multiple regression analysis are shown in Table 3. The analysis included 10 variables into the final model (R^2^ = 0.110): sex, monthly household income, education level, living area, currently working, victim of armed conflict, type of disability, frequency of communication with neighbors or friends, and usual participation in community organization activities.

The significant variables were monthly household income (β = 0.45, *p* = 0.028), education level (β = 0.65, *p* = 0.048), currently working (β = 1.00, *p* = 0.017), type of disability (β = −1.17, *p* = 0.002), frequency of communication with neighbors or friends (β = 0.53, *p* = 0.013), and participation in community organization activities (β = 0.89, *p* = 0.019).

## 4. Discussion

Our study identified that determinants of participants’ higher level of self-esteem were frequent communication with neighbors or friends, active participation in community activities, higher education level, having a current job, and higher monthly household income.

A previous study reported that the mean RSES of the general Colombian population without any disabilities was 34.2 [30]. In the present study, the mean score of persons with disabilities was much lower (*M* = 28.8 [SD = 4.5]). This result coincided with a previous study showing that persons with disabilities have lower self-esteem than peers without disabilities [13]. RSES score was 33.9 for the persons with spinal cord injury living in Neiva, one of the provincial cities in Colombia [31]. Compared to persons with disabilities in provincial cities, the participants in the current study had lower RSES scores. The persons with disabilities in Neiva did not from the control group without disabilities (32.2) [31]. Furthermore, they could access more education and rehabilitation programs. The current study conducted in rural areas found that education might have contributed significantly to higher self-esteem; however, opportunities for education might be more limited than in a previous study [31]. A large opportunity gap for accessing education or rehabilitation services might exist between the provincial and local municipalities. This gap may have influenced the level of self-esteem in both studies.

In the present study, self-esteem was also associated with the following factors: more communication with neighbors and friends, active participation in the community organization, having a job, and monthly household income. These findings are similar to those of previous studies [13,15,16,20,22]. Therefore, this study strongly suggests that the promotion of active social participation among persons with disabilities would be important to achieve higher self-esteem. To fortify and respect the position of persons with disabilities in the context of respecting human rights, we should promote this even more in developing countries as well. However, the findings must be interpreted carefully because this study has some limitations. One limitation is that the data were collected from only two local areas selected by the JICA Project. The sociodemographic status and social participation of people with disabilities were different in two local municipalities (Table S2). This limited the generalizability of the findings. Furthermore, this study could not reflect all types of disability, because we excluded those with communication difficulties and those requiring assistance to answer the questions (Table S3). Second, the present study did not include a control group, such as persons without disabilities in the same area. Third, we did not include variables such as the primary illness of each participant, which possibly affected the results. These variables might be affected by self-esteem and life satisfaction. Fourth, because this study was a cross-sectional study, it was not possible to infer a cause-and-effect relationship between social participation and self-esteem. Longitudinal or experimental studies are necessary to clarify causality, and it may feature a more effective support program.

Despite these limitations, the present study findings are worthy and valuable for the following reasons. In Colombia, there have been no full surveys focusing on persons with disabilities in rural areas, especially in conflict-affected areas. Moreover, no study has directly interviewed a large number of persons with disabilities. This study focused on a normally hidden population that is hard to access. In particular, people with disabilities living in local municipalities affected by armed conflict are difficult to invite to field-based studies. Furthermore, the data were collected from persons with various types of disability who could answer the questionnaire by themselves. We believe that these findings from Colombia could contribute to disseminating useful information that social participation is important to improve the self-esteem of persons with disabilities living in similar conditions. Furthermore, the findings suggest the importance of promoting a national reconstruction program for social inclusion (involving persons with disabilities) in Colombia, emphasized in the Goal 16 of the Sustainable Development Goals (SDGs), which is to promote a just, peaceful, and inclusive society [32].

## 5. Conclusions

This study focused on persons with various types of disability living in local municipalities affected by armed conflict in Colombia. Frequent communication in one’s own community, higher education, and having a job were identified as determinants of higher self-esteem in persons with disabilities in local municipalities affected by armed conflict. Promoting inclusive reconstruction programs to support persons with disabilities affected by armed conflict is extremely crucial in global health issues. In particular, this study strongly exhibited that more social inclusion of people with disabilities may promote global development, global peace, and order. This is consistent with Goal 16 of the SDGs, which is to promote a just, peaceful, and inclusive society. As a next step, it would be important to evaluate the effectiveness of programs and activities developed as a result of the findings and the extent to which Goal 16 is being achieved.

## Figures and Tables

**Table 1:** Sociodemographic characteristics and social participation of the participants (*N* = 579).

Variable	Mean	SD	*n*	%
Age (years)	51.9	16.6		
Sex				
Female			260	44.9
Monthly Household Income ^1^				
None [0 Colombian pesos (COP)			17	2.9
Low [<100,001 COP (approximately 32 US dollars)]			106	18.3
Lower middle [<350,001 COP (approximately 113 US dollars)]			262	45.3
Upper middle [<700,001 COP (approximately 226 US dollars)]			150	25.9
High [700,001 COP (approximately 226 US dollars)]			44	7.6
Education Level ^2^				
None or pre-school level			79	13.6
Basic primary [1–5 years]			374	64.6
Secondary school and above [6 years or more]			126	21.8
Have Reading Ability (including Braille)			448	77.4
Have Writing Ability (including Braille)			456	78.8
Residential Area				
Urban			234	40.4
Rural			345	59.6
Currently Working				
Yes			236	40.8
Victim of Armed Conflict				
Yes			457	78.9
Type of Disability				
Physical			248	42.8
Hearing			18	3.1
Visual			69	11.9
Intellectual			37	6.4
Psychosocial			61	10.5
Multiple			104	18.0
Do not know			42	7.3
Frequency of Communication with Family Members				
Every day			482	83.3
Some days a week			9	1.6
Occasionally or do not communicate			88	15.2
Frequency of Communication with Neighbors or Friends				
Every day			178	30.7
Some days a week			96	16.6
Occasionally or not communicate			305	52.7
Go out usually			399	68.9
Participation in Activities of the Community Organization				
Yes			225	38.9

^1^ Exchange rate at 2015. ^2^ Education system in Colombia has 5 years in primary education, 4 years in pre-secondary education, 2 years in post-secondary education, and 5 years in higher education.

**Table 2:** Participants’ scores on the Rosenberg Self Esteem Scale (RSES) by socioeconomic status and social participation based on the univariate regression analysis (*N* = 579).

Variables	RSES^1^ Score [Mean (SD)]	Univariate Regression			
		β	95% CI		*p*
Total	28.8 (4.5)				
Age, years (Higher)		−0.21	−0.70	0.27	0.383
<40	29.0 (5.0)				
40–60	28.9 (4.3)				
>60	28.6 (4.5)				
Sex (Female [1] vs. Male [0])		0.16	−0.58	0.91	0.667
Male	28.7 (4.6)				
Female	28.9 (4.5)				
Monthly Household Income (Higher)		0.65	0.25	1.06	0.002
None [0 Colombian pesos (COP)]	26.4 (4.4)				
Low [<100,001 COP (approximately 32 US dollars)]	28.6 (4.6)				
Lower middle [<350,001 COP (approximately 113 US dollars)]	28.5 (4.4)				
Upper middle [<700,001 COP (approximately 226 US dollars)]	29.1 (4.7)				
High [700,001 COP (approximately 226 US dollars)]	30.7 (4.2)				
Education Level (Higher)		1.27	0.65	1.89	<0.001
None or pre-school level	27.3 (4.5)				
Basic primary	28.7 (4.4)				
Secondary school and above	29.9 (4.6)				
Have Reading Ability (including Braille) (Yes [1] vs. No [0])		1.19	0.31	2.08	0.008
Yes	29.1 (4.6)				
No	27.9 (4.2)				
Have Writing Ability (including Braille) (Yes [1] vs. No [0])		1.43	0.53	2.33	0.002
Yes	29.1 (4.6)				
No	27.7 (4.1)				
Residential Area (Urban [1] vs. Rural [0])		1.01	0.26	1.76	0.011
Urban	29.4 (4.3)				
Rural	28.4 (4.7)				
Currently Working (Yes [1] vs. No [0])		1.00	0.25	1.75	0.009
Yes	29.4 (4.4)				
No	28.4 (4.6)				
Victim of Armed Conflict (No [1] vs. Yes [0])		−1.05	−1.95	−0.14	0.024
Yes	29.0 (4.5)				
No	28.0 (4.5)				
Type of Disability (others [1] vs. physical disability [0])		−1.25	−1.99	−0.51	0.001
Physical	29.5 (4.6)				
Others	28.3 (4.5)				
Frequency of Communication with Family Members (Higher)		0.20	−0.32	0.71	0.449
Every day	28.9 (4.6)				
Some days a week	30.0 (4.2)				
Occasionally or do not communicate	28.4 (4.5)				
Frequency of Communication with Neighbors or Friends (Higher)		0.85	0.44	1.26	<0.001
Every day	30.0 (4.5)				
Some days a week	28.5 (4.4)				
Occasionally or do not communicate	28.2 (4.5)				
Go out Usually (Yes [1] vs. No [0])		1.50	0.71	2.29	<0.001
Yes	29.3 (4.2)				
No	27.8 (5.0)				
Participation in Activities of the Community Organization (Yes [1] vs. No [0])		1.20	0.44	1.95	0.002
Yes	29.5 (4.4)				
No	28.3 (4.6)				

^1^ The project team translated the RSES into Spanish and modified the words to reflect the Colombian rural situation (RSES Cronbach’s α = 0.73).

**Table 3:** Multivariate regression analysis on the association of socioeconomic status and social participation with self-esteem (*N* = 579) (R^2^ = 0.110).

Variable	β	95% CI		*p*
Age (Higher)	-	-	-	-
Sex (Female (1) vs. Male (0))	0.56	−0.23	1.34	0.165
Monthly Household Income (Higher)	0.45	0.05	0.86	0.028
Education Level (Higher)	0.65	0.01	1.29	0.048
Reading Ability (Yes (1) vs. No (0))	-	-	-	-
Writing Ability (Yes (1) vs. No (0))	-	-	-	-
Residential Area (Urban (1) vs. Rural (0))	0.67	−0.14	1.49	0.106
Currently Working (Yes (1) vs. No (0))	1.00	0.18	1.83	0.017
Victim of Armed Conflict (No (1) vs. Yes (0))	−0.65	−1.55	0.26	0.161
Type of Disability (others (1) vs. physical disability (0))	−1.17	−1.89	−0.45	0.002
Frequency of Communication with Family Members (Higher)	-	-	-	-
Frequency of Communication with Neighbors or Friends (Higher)	0.53	0.11	0.95	0.013
Go out Usually (Yes (1) vs. No (0))	0.71	−0.11	1.54	0.090
Participation in Activities of the Community Organization (Yes (1) vs. No (0))	0.89	0.14	1.64	0.019

“-” were not selected by stepwise variable selection method (inclusion criterion *p* ≤ 0.20, exclusion criterion *p >* 0.20).

## Data Availability

No new data were created or analyzed in this study. Data sharing is not applicable to this article.

## Supplementary Materials

The following are available at https://www.mdpi.com/article/10.3390/ijerph18084328/s1, Table S1: Socioeconomic status and social participation items with their response options, Table S2: Comparison of sociodemographic status and social participation between El Carmen de Chucuri (*n* = 252) and Granada (*n* = 327), Table S3: Comparison of persons with disabilities (PwDs) who completed the Rosenberg Self-Esteem Scale (RSES) with PwDs who did not complete the RSES (N = 783).
