# Supplementary material for: Self-Esteem, Socioeconomic Status and Social Participation of Persons with Disabilities Living in Areas Affected by Armed Conflict in Colombia
Source: Int J Environ Res Public Health. 2021 Apr 19;18(8):4328. doi: 10.3390/ijerph18084328 (PMC8073117; doi:10.3390/ijerph18084328)
Supplement: Supplementary file 1 [file ijerph-18-04328-s001.zip › ijerph-1137064 supplementary final_ty.pdf]

Supplementary Materials

Table S1. Socioeconomic status and social participation items with their response options.

Items	Response options
Age	years
Sex	Male/Female
Monthly household income [Colombian peso [1] (US dollar)]	None [0 Colombian pesos (COP) Low [<100,001 COP (about 32 US dollars)]/Lower middle [<350,001 COP (about 113 US dollars)]/Upper middle [<700,001 COP (about 226 US dollars)]/High [700,001 COP (about 226 US dollars)]
Education Level [2]	None or pre-school level/Basic primary/Secondary school or above
Have reading ability (including Braille)	Yes/No
Have writing ability (including Braille)	Yes/No
Residential area	Urban/Rural
Current work	Yes/No
Victim of armed conflict	Yes/No
Type of disability	Physical disability/Hearing disability/Visual disability/ Intellectual disability/Psychosocial disability/Do not know
Frequency of communication with family members	Every day/Some days a week/Occasionally or not communicate
Frequency of communication with neighbors or friends	Every day/Some days a week/Occasionally or not communicate
Going out usually from home	Yes/No
Participation in activities of community organization	Yes/No

[1] Exchange rate at 2015. [2] Education system in Colombia has 5 years in primary education, 4 years in pre-secondary education, 2 years in post-secondary education, and 5 years in higher education.

Table S2. Comparison of sociodemographic status and social participation between El Carmen de Chucuri (n = 252) and Granada (n = 327).

**Variables**	**El Carmen de Chucuri**		**GRANADA**		***P*** [3]
	n	**%**	***n***	**%**	
Age (mean [SD])	[M = 49.0(16.5)]		[M = 54.1(16.3)]		<0.001[4]
Sex					0.027
Female	100	39.7	160	48.9	
Monthly Household Income [1]					0.008
None [0 Colombian pesos (COP) (0 US dollar)]	14	5.6	3	0.9	
Low [<100,001 COP (approximately 32 US dollars)]	42	16.7	64	19.6	-
Lower middle [<350,001 COP (approximately 113 US dollars)]	108	42.9	154	47.1	
Upper middle [<700,001 COP (approximately 226 US dollars)]	72	28.6	78	23.9	
High [700,001 COP (approximately 226 US dollars)]	16	6.4	28	8.6	
Education Level [2]					<0.001
None or pre-school level	59	23.4	20	6.1	
Basic primary [1–5 years]	140	55.6	234	71.6	
Secondary School and above [6 years or more]	53	21.0	73	22.3	
Have reading ability (including Braille)	190	75.4	258	78.9	0.318
Have writing ability (including Braille)	193	76.6	263	80.4	0.263
Residential Area					<0.001
Urban	40	15.9	194	59.3	
Currently Working					0.032
Yes	120	47.6	116	35.5	
Victim of armed conflict					<0.001
Yes	136	54.0	321	98.2	
Type of disability					<0.001
Physical	104	41.3	144	44.0	
Hearing	9	3.6	9	2.8	
Visual	28	11.1	41	12.5	
Intellectual	13	5.2	24	7.3	
Psychosocial	21	8.3	40	12.2	
Multiple	38	15.1	66	20.2	
Do not know	39	15.5	3	0.9	
Frequency of communication with family members					0.743
Every day	213	84.52	269	82.26	
Some days a week	4	1.59	5	1.53	
Occasionally or do not communicate	35	13.89	53	16.21	
Frequency of communication with neighbors or friends					0.963
Every day	77	30.6	101	30.9	
Some days a week	43	17.1	53	16.2	
Occasionally or not communicate	132	52.4	173	52.9	
Go out usually	162	64.3	237	72.5	0.035
Participation in activities of the community organization	83	32.9	142	43.4	0.010
Rosenberg Self Esteem Scale (mean [SD])	[M = 28.3(4.9)]		[M = 29.2(4.2)]		0.015[4]

[1] Exchange rate at 2015. [2] Education system in Colombia has 5 years in primary education, 4 years in pre-secondary education, 2 years in post-secondary education and 5 years in higher education. .[3] χ squared test. [4] t-test.

Table S3. Comparison of persons with disabilities (PwDs) who completed the Rosenberg Self-Esteem Scale (RSES) with PwDs who did not complete the RSES (N = 783).

Variable	PwDs who completed RSES (n = 579)		PwDs who did not complete RSES (n = 204)		p [3]
	n	%	n	%	
Age [mean(SD)]	[M = 51.9(16.6)]		[M = 47.2(18.6)]		0.002 [4]
Sex					0.772
Female	260	44.9	94	46.1	
Monthly Income of the Household (Colombian peso [1])		-			0.947
None [0 Colombian pesos (COP)]	17	2.9	7	3.4	
Low [<100,001 COP (about 32 US dollars)]	106	18.3	41	20.1	
Lower middle [<350,001 COP (about 113 US dollars)]	262	45.3	86	42.2	
Upper middle [<700,001 COP (about 226 US dollars)]	150	25.9	54	26.5	
High [700,001 COP (about 226 US dollars)]	44	7.6	16	7.8	
Education Level [2]					<0.001
None or pre-school level	79	13.6	100	49.0	
Basic primary	374	64.6	96	47.1	
Secondary school and above	126	21.8	8	3.9	
Have Reading Ability (including Braille)	448	77.4	52	25.5	<0.001
Have Writing Ability (including Braille)	456	78.8	51	25.0	<0.001
Living Area					0.577
Urban	234	40.4	87	42.7	
Rural	345	59.6	117	57.4	
Current Work					<0.001
Yes	236	40.8	39	19.1	
Victim of Armed Conflict					0.045
Yes	457	78.9	147	72.1	
Type of Disability					<0.001
Physical	248	42.8	11	5.4	
Hearing	18	3.1	19	9.3	
Visual	69	11.9	3	1.5	
Intellectual	37	6.4	51	25.0	
Psychosocial	61	10.5	48	23.5	
Multiple disabilities	104	18.0	56	27.5	
Do not know	42	7.3	16	7.8	
Can you hear the voice or sounds?					<0.001
Cannot do at all	9	1.55	21	10.29	
A lot of difficulty	40	6.91	24	11.76	
Some difficulty	72	12.44	26	12.75	
No difficulty	458	79.1	133	65.2	
Can you speak or talk?					<0.001
Cannot do at all	8	1.38	41	20.1	
A lot of difficulty	31	5.35	62	30.39	
Some difficulty	47	8.12	46	22.55	
No difficulty	493	85.15	55	26.96	
Can you see from near, far, or around?					<0.001
Cannot do at all	11	1.9	6	2.94	
A lot of difficulty	151	26.08	35	17.16	
Some difficulty	207	35.75	30	14.71	
No difficulty	210	36.27	133	65.2	
Can you move your body, walk?					<0.001
Cannot do at all	18	3.11	12	5.88	
A lot of difficulty	184	31.78	32	15.69	

Some difficulty	158	27.29	49	24.02	
No difficulty	219	37.82	111	54.41	
Can you grab or move objects with your hands?					0.001
Cannot do at all	6	1.04	8	3.92	
A lot of difficulty	86	14.85	20	9.8	
Some difficulty	116	20.03	26	12.75	
No difficulty	371	64.08	150	73.53	
Can you learn, remember, make decisions?					<0.001
Cannot do at all	19	3.28	87	42.65	
A lot of difficulty	70	12.09	66	32.35	
Some difficulty	130	22.45	31	15.2	
No difficulty	360	62.18	20	9.8	
Can you eat, dress, bathe by yourself?					<0.001
Cannot do at all	6	1.04	23	11.27	
A lot of difficulty	48	8.29	27	13.24	
Some difficulty	99	17.1	27	13.24	
No difficulty	426	73.58	127	62.25	
Can you relate or interact with others?					<0.001
Cannot do at all	6	1.04	33	16.18	
A lot of difficulty	38	6.56	59	28.92	
Some difficulty	70	12.09	57	27.94	
No difficulty	465	80.31	55	26.96	
Can you perform everyday tasks without having heart or breathing problems?					<0.001
Cannot do at all	12	2.07	20	9.8	
A lot of difficulty	53	9.15	24	11.76	
Some difficulty	144	24.87	39	19.12	
No difficulty	370	63.9	121	59.31	
Frequency of Communication with Neighbors or Friends					0.104
Every day	178	30.7	52	25.49	
Some days a week	96	16.6	27	13.24	
Occasionally or not communicate	305	52.7	125	61.27	
Going out usually	399	68.9	112	54.9	<0.001
Participation in Activities of Community Organization					<0.001
Yes	225	38.9	38	18.6	

[1] Exchange rate at 2015. [2] Education system in Colombia has 5 years in primary education, 4 years in pre-secondary education, 2 years in post-secondary education and 5 years in higher education.[3] χ squared test. [4] t-test.
